# 

**Published:** 1887-12-31

**Authors:** 


### NOTICES.

The scale of charges for small prepaid Advertisements—Situations Wanted, Situations Vacant, &c., &c., is as follows:—

| | s. | d. |
|---|---|---|
| One insertion of three lines ........................ | 1 | 0 |
| Three " " " ........................ | 2 | 6 |
| Per line afterwards ........................ | 0 | 6 |

*A line averages ten words.*

All copy for Advertisements must be sent to A. P. Watt, 2, Paternoster Square, E.C., by first post on the Tuesday *preceding* publication.

**All communications respecting editorial matters** should be addressed to the Editor, 38, Old Jewry, Cheapside, E.C.

### THE STUDENTS' COLUMN.

### UNIVERSITY OF LONDON.

B.S. EXAMINATION.

EXAMINATION FOR HONOURS.

SURGERY.—First Class: Roughton, Edmund W., M.D. (Scholarship and Gold Medal), St. Bartholomew's; Tubby, Alfred Herbert (Gold Medal), Guy's; Carless, Albert,* King's College; Braddon, William Leonard, Guy's; Brown, Herbert Henry, University College. Second Class: Holder, Sydney Ernest, University College; Wynter, Walter Essex, St. Bartholomew's and Middlesex; Clarke, William Frederick, Guy's; Flemming, Percy, University College.

* Obtained the number of marks qualifying for a Gold Medal.

N.B.—The bracket denotes equality of merit.

### QUESTIONS AND ANSWERS.

WILL "Inquirer" (Northampton) send her name and address, not necessarily for publication?

### Amusements with Profit for all Ages.

**Rules.**—All answers must be addressed to the Prize Editor, The Hospital, 38, Old Jewry, Cheapside, London, E.C., not later than the *first* post on Thursday next. The full name and address must in all cases be given, but a *nom de plume* may be added for publication. Only one side of the paper must be written on.

### FOR MEN AND WOMEN.

### Work Competition.

I. A PRIZE OF THREE GUINEAS will be given to the competitor who sends in the best specimen of painting, which may take the form of a picture, panel, screen, flower-pots, etc., or illuminations.

II. TWO GUINEAS will be given for the best dressed doll or other piece of work suitable for presentation to the inmates of the hospitals.

III. ONE GUINEA will be given for the best scrap-book.

All Contributions for this competition must be sent in by January 7th, 1888, addressed "Work Competition," care of the Editor, The Lodge, Porchester Square, London, W.

### Acrostic Competition.

A PRIZE OF ONE GUINEA will be given to the competitor who gains the highest number of marks for best original acrostics during the ensuing quarter. All acrostics sent in for this competition will be credited according to merit, twelve marks being the highest score given for one acrostic.

A PRIZE of HALF-A-GUINEA to be given to the competitor who gains the highest score for solution of acrostics set, including the one inserted on November 26th. One mark only will be given to the competitors who solve all the lights of each acrostic.

These competitions to alternate weekly.

This week credit will be given for No. 3 Double Original Acrostic in verse.

### Results of No. 2 Original Acrostic.

Brownie (12), Pasteur (12), Ellice (12), A. M. E. (12), Mater (11), Padre (11), K. M. (10), Greta (8), Condorcet (7.)

### II. Word and Literary Competition.

Commenced November 5th, 1887; ends January 28th, 1888.

Three prizes of one guinea, 15*s*. and 10*s*. 6*d*. will be given each quarter to the three competitors who obtain the highest number of marks: (1) by making the largest number of words derived from the word or phrase set for anatomical dissection; or (2) for the best answers to (6); or (3) by (1) and (2) combined.

(A) THE WORD COMPETITION.

We shall give the newspapers for dissection during this quarter, that for this week being

"The Queen."

Observe.—Proper names, abbreviations, foreign words, words of less than four letters and repetitions are barred. Nuttall's *Standard* dictionary only to be used.

### Results of Seventh Week.

The Globe.—Ellice (33), Reldas (33), Gretta (33), Brisbane (29), Skye (28), Kath (25), K. M. (24), Condorcet (22), Brownie (20).

### THE THEATRES.

The merry plays presented with so much success by Mr. Hawtrey are now succeeded by a melodrama of the deepest dye at the Globe Theatre, which has been redecorated throughout for the reception of Mr. Wilson Barrett, after his long absence in America and the country. His reception was a most cordial one, and he had to bow many times before the applause with which he was greeted on his first appearance subsided. He has himself joined with Mr. G. R. Sims in the authorship of the *Golden Ladder*, with which he commences his new tenancy at the Globe.

It is a long and complicated play, embracing many situations for which there is not in the new and smaller theatre so much scope as the fine stage of the Princess's offered for telling situations; but wonders have been worked in the preparation for scenic effect and changes introduced in which the Globe stage has been completely metamorphosed. The early scenes are laid at Kingston-on-Thames, and tell us of the probable and speedy ruin of Mr. John Grant, banker, by his cashier, Severn, who is enraged at the refusal of Miss Grant to listen to his offers of love. The hero, the Rev. Frank Thornhill, comes now to the rescue, and with a fortune he has made in Madagascar pays the claims against the old banker, saves the credit of the family name, and marries Miss Grant, his old love. The second act introduces us to Madagascar, where Mr. Thornhill is working as English missionary amongst the Hovas, and where he incurs the enmity of the French—for the time is the eve of the French troubles in the island. In spite of his efforts to maintain peace, the evil genius of the play, Michael Severn, with his colleague in villainy, M. Peranza, prompts Rao to poison the wine which Mr. Thornhlil provides for the French. In Act III. we are taken back to England, and find the villains once more trading together as Keith and Co. In the same act there is some genuinely comic business in the Peckaby's Cottage at Hampstead, which is, in our mind, much the best part of the very long play. The latter part of the act is devoted to the tragedy which leads to Act IV., wherein we have heartrending scenes in the strongest colours, arising from the imprisonment of Mrs. Thornhill (née Lilian Grant), terminating with the escape of Mrs. Thornhill from the prison. However, in Act V.—for there are five acts, and they take three hours and a-half to play—all ends well; the villains are unmasked by their accomplice, Mrs. Thornhill is pardoned, and the Rev. Frank recovers his property.

Mr. Barrett himself takes the part of the Rev Frank Thornhill, and plays it with dignity, but his objectionable manner of speech is more pronounced than before he went away, and he appears to rely more than ever upon poses and the beautifying effect of the limelight, without, however, the same success as at the Princess's. Mr. Barrett is not pleasing as an actor, but he has a marked and distinct individuality, and, such being the case, must always have a large following.

The task of playing the heroine, Lilian Grant, devolves on Miss Eastlake, who plays in her usual well-known style. With Mr. Barrett she makes a very strong scene out of the interview in the prison. The villains of the piece—there are many of them—are very well portrayed by Messrs. Melford, Cliffe, and Hudson, and the minor characters—there are thirty altogether—are generally well filled.

Though we have not yet mentioned Mr George Barrett and Mrs. Leigh, we have only postponed doing so that we might speak of them with more effect. If the play succeeds, they will have contributed, with the assistance of Miss Phœbe Carlo, in no small degree to this success. The Peckaby family complete is one of the funniest we have seen, and both Mr. and Mrs. Peckaby meet with marked success when they show their affection for and devotion to the Rev. Mr. Thornhill and his wife, who receive nothing else but kicks from the rest of their friends. Mr. G. R. Sims' melodramas please many, and in Mr. Wilson Barrett he has an exceedingly strong exponent of the characters he loves to draw, and the two together are sure to draw large houses.

A notable event of last week was the opening of the doors of the Empire Theatre, and so auspicious was the event that there is every hope of the theatre having at last been put to a paying purpose. The license for music and dancing has only been granted after a terribly long and severe struggle, but now, as the new palace, for it is little else than a palace, so luxurious are all the appointments and fittings, is under the powerful management of Mr. Augustus Harris and Mr. George Edwardes, the Alhambra and the Pavilion will have to look to their laurels. M. Hervé directs the orchestra, and the first ballet, "Dilara," is extremely pretty and bound to fill the house nightly. The second ballet, devoted to the sports of England, is also likely to prove very popular.

Before concluding this article we may mention the improved prospects of the Royal Aquarium, where the marvellous powers of Professor Roche over his pack of fifteen Russian wolves are being shown, as well as the Tunisian beauty La Belle Fatma, who has certainly gone off very much since she was travelling round the French fairs two years ago. Many other good things worth seeing make up a most attractive programme, where an afternoon might be passed with pleasure.

### THE CHILDREN'S CORNER.

PRIZES.

FOR MOST CORRECT ANSWERS TO PUZZLES.

This Commenced October 1st, 1887.

One Pound a Month.

FOR BEST LITTLE LETTERS.

Ten Shillings per Quarter.

A PRIZE OF THREE GUINEAS to the Competitor who shall have sent in the largest number of correct or best answers during the twelve months.

### Puzzles—Fifth Week.

No. 17. Enigma.

I am a word of three letters:
Behead me and you'll triple me;
Behead again, you'll make me ten.
My whole will just your state express,
Unless the word you quickly guess.

No. 18. Double Acrostic.

In the silence of the night,
No one hears my footfall light;
But with dawn the children peeping.
Find I've been while they were sleeping.

1. This law forbids a Queen to reign,
2. O'er you I watch in joy and pain;
3. Hark to my roar! Great as my fame,
4. Part of a plant, from Holland it came,
5. My fifth and last is minus a name.

No. 20. French Puzzle Sentence.

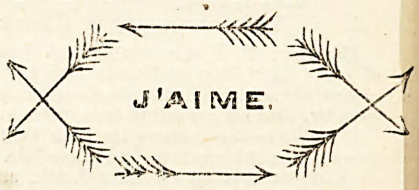

Who can explain this French puzzle?

No. 13. Biographical Charade.—My *first* denotes a stage in vegetable growth; my *second* a piece of ground generally cultivated; my *whole* an English Poet.

### Notices to Correspondents.

*Mount Edgcumbe*—H. E.—Puzzles received too late to be credited.

### Results of Third Week Puzzles.

No. 9. Double Acrostic.

C urlin G

H ampe R

R etir E

I nvit E

S piri T

T ahit I

M usicia N

A wnin G

S ucces S

No. 10. Numerical Proverb.—Money makes the mare to go.

No. 11. Hem-i-sphere (Hemi').

No. 12. Geographical Charade. — Manchester=Manchester.

All right:—Pepita, Gem, Scrooge, Tim. Three right:—A. G. S. McCulloch, Holmes, Squeaker, Military Cadet, Skye, Ossian, Plummy Fluff, A. C. K., Jumps, Excelsior. Two right:—Poodle, Betty Burke, Spider, M. S. W.

### Letters.

The results of the last Children's Quarterly Letter Competition will be published next week. In future this competition will be discontinued, and we shall publish instead letters from friends acros the seas.

**Notice to all Correspondents.**—N.B.—All letters referring to this page, which do not arrive at 38, Old Jewry, Cheapside, London, E.C. by the *first post on Thursdays*, and are not addressed PRIZE EDITOR, will in future be disqualified and disregarded.

No one will be permitted to win the Monthly or Quarterly Prizes twice in any one year, the annual prizes being offered especially to prevent this.

